# Comparison of efficacy and safety between PD-1 inhibitors and PD-L1 inhibitors plus platinum-etoposide as first-line treatment for extensive-stage small-cell lung cancer: a multicenter, real-world analysis

**DOI:** 10.1186/s12885-023-11709-1

**Published:** 2023-12-06

**Authors:** Yanrong Wang, Lingling Li, Jia Hu, Yan Zhao, Huan Yan, Ming Gao, Xuejiao Yang, Xia Zhang, Junxun Ma, Guanghai Dai

**Affiliations:** 1 Department of Medical Oncology, The First Medical Center, Chinese PLA General Hospital, Beijing, China; 2 Chinese PLA Medical School, Beijing, China; 3 Department of Medical Oncology, The Fifth Medical Center, Chinese PLA General Hospital, Beijing, China; 4 Department of Medical Oncology, The Seventh Medical Center, Chinese PLA General Hospital, Beijing, China; 5 School of Medicine, Nankai University, Tianjin, China

**Keywords:** Real-world data, Small-cell lung cancer, PD-1 inhibitor, PD-L1 inhibitor, First-line treatment, Efficacy, Prognosis

## Abstract

**Background:**

Immunotherapy in combination with platinum-etoposide (EP) chemotherapy has been approved as a first-line treatment for extensive-stage small cell lung cancer (ES-SCLC). However, real-world (RW) data regarding the use of immune checkpoint inhibitors (ICIs) in ES-SCLC are lacking. We aimed to assess the differences between programmed death protein 1 (PD-1) inhibitors and programmed death ligand 1 (PD-L1) inhibitors, both in conjunction with EP chemotherapy, as first-line treatment for ES SCLC.

**Methods:**

We conducted a real-world, multicenter, retrospective cohort, controlled study to compare the prognosis, efficacy, and safety of PD-1 and PD-L1 inhibitors in ES-SCLC patients when used along with chemotherapy. Each patient received up to six cycles of etoposide, carboplatin, or cisplatin combined with ICI drugs, including PD-1 and PD-L1 inhibitors. The primary endpoints were investigator-assessed progression-free survival (PFS) and overall survival (OS). The secondary endpoints were the investigator-assessed objective response rate (ORR) and disease control rate (DCR) according to the Response Evaluation Criteria in Solid Tumors (RECIST, version 1.1).

**Results:**

Between January 2017 and December 2021, 194 patients with ES-SCLC from three clinical centers in a PLA general hospital were included in our study, including 93 patients in the PD-1 group and 101 patients in the PD-L1 group. At the time of data cutoff, progression-free survival in the PD-1 group (median PFS, 6.8 months; 95% CI, 5.3–8.1) was similar to that in the PD-L1 group (median PFS, 6.4 months; 95% CI, 5.5–7.5); the stratified hazard ratio for PFS was 1.12 (95% CI, 0.83–1.53; P = 0.452). The median OS was similar in the PD-1 and PD-L1 groups (15.8 m vs. 17.7 m, P = 0.566); the hazard ratio was 0.90 (95% CI, 0.62–1.30, P = 0.566). The two groups had comparable investigator-assessed confirmed objective response rates (ORR) (76.3% vs. 76.2%). Adverse effect (AE)-related discontinuation occurred in 4 (4.3%) patients in the PD-1 group and 2 (2.0%) patients in the PD-L1 group. Deaths due to AEs of any cause occurred in 2 (2.2%) patients in the PD-1 inhibitor group and 1 (1.0%) patient in the PD-L1 inhibitor group.

**Conclusions:**

Our research revealed that there were no significant differences in efficacy or prognosis between PD-1 inhibitor + EP chemotherapy and PD-L1 inhibitor + EP chemotherapy. The two groups seemed to have comparable safety profiles, but the number of discontinuation or death events was too small to draw a firm conclusion.

**Supplementary Information:**

The online version contains supplementary material available at 10.1186/s12885-023-11709-1.

## Introduction

Small-cell lung cancer (SCLC), accounting for approximately 15% of all diagnosed cases of lung cancer, is an aggressive neuroendocrine malignancy strongly associated with rapid proliferation, a high growth fraction, and the early development of widespread metastases [1, 2]. Due to the aggressive nature described above, nearly 70% of patients already had distant metastasis at first diagnosis, which was defined as extensive-stage small cell lung cancer (ES-SCLC). The prognosis of ES-SCLC is poor, with a median overall survival of approximately 10 months, and the five-year survival rate remains at approximately 6–7% after diagnosis [3–5]. Despite comprehensive research on the therapeutic innovations of ES-SCLC, the etoposide-platinum (EP) chemotherapeutic regimen has been the standard first-line systemic anticancer therapy for SCLC for more than three decades [2, 6, 7]. Although first-line treatment response rates are up to 78% for ES-SCLC patients, responses are not durable, and most patients experience relapse within 6 months [4, 5].

With the strong immunogenic features of SCLC cells and the stimulated release of tumor antigens by chemotherapy, the combination of immune checkpoint inhibitors (ICIs) and chemotherapy is highly anticipated. As programmed death protein 1 (PD-1) inhibitors, nivolumab and pembrolizumab monotherapy showed a modest response in certain subgroups in SCLC initial studies, including the Keynote-028, Checkmate-032, and Keynote-158 trials, leading to their clinical use in the later-line treatment of metastatic ES-SCLC [8–10]. The ASTRUM-005 randomized clinical trial (RCT) showed that another PD-1 inhibitor, serplulimab plus chemotherapy, significantly improved overall survival compared with chemotherapy alone in patients with previously untreated extensive-stage SCLC [11]. Serplulimab was approved by the National Medical Products Administration (NMPA) in combination with chemotherapy for first-line treatment of ES-SCLC, reshaping the first-line treatment landscape of ES-SCLC in China.

Furthermore, the programmed death ligand 1 (PD-L1) pathway has demonstrated clinical activity as a first-line treatment in patients with ES-SCLC. As a multinational, phase 3, double-blind, randomized, placebo-controlled trial, IMpower133 was the first successful phase III trial that demonstrated the efficacy and safety of atezolizumab in combination with carboplatin-etoposide as first-line treatment in patients with extensive-stage small-cell lung cancer [12]. In phase 3, randomized, open-label CASPIAN study, another PD-L1 inhibitor durvalumab plus platinum-etoposide as first-line treatment for ES-SCLC patients resulted in consistent and durable clinical benefit across overall survival (OS), progression-free survival (PFS), and objective response compared with a clinically relevant control group [13]. Based on the abovementioned findings, the Food and Drug Administration (FDA) approved PD-L1 inhibitors, including atezolizumab and durvalumab, in combination with chemotherapy as the first-line regimen for treatment-naive patients with ES-SCLC in 2019 and 2020, respectively. The Keynote-604 study was another phase III clinical trial that suggested that pembrolizumab plus EP significantly improved PFS but not OS compared to placebo plus EP as first-line therapy for patients with ES-SCLC [14].

Despite these encouraging findings of clinical trials, because randomized controlled trials have strict inclusion and exclusion criteria, patients with poor performance, active brain metastasis, a history of autoimmune disease, or planned consolidation chest radiotherapy were excluded. In addition, in the IMpower133, CASPIAN, and KEYNOTE 604 studies, Asian patients accounted for only 17%, 14%, and 22.8%, respectively.

Differences between the clinical performance of PD-L1 inhibitors and PD-1 inhibitors have been reported in several studies. The underlying mechanisms are still not fully elucidated, but one possible reason could be the interaction between PD-1 and PD-L2, which may also inhibit T-cell activation. PD-1 inhibitors can block the binding of PD-1 with both PD-L1 and PD-L2, while PD-L1 inhibitors only inhibit the binding of PD-1 with PD-L1. Therefore, when treated with a PD-L1 inhibitor, tumors may evade anti-tumor immune responses through the PD-1/PD-L2 axis [15–17]. Another possible explanation is that PD-L1 binds two receptors, PD-1 and B7.1 (CD 80). B7.1 on tumor-associated dendritic cells (DCs) is a key co-stimulatory molecule that enhances T cell activation through the interaction with B7.1/CD 28. Therefore, PD-L1 inhibitors exert a greater effect than PD-1 inhibitors by blocking PD-L1 on DCs, which in turn relieves the inhibition of B7.1 and further restores DC function to facilitate the initiation of anti-cancer T cell immunity. However, for SCLC, our results indicate that there is no significant difference in overall survival (OS) and progression-free survival (PFS) between PD-L1 + Chemo and PD-1 + Chemo [17–19]. A potential explanation could be that PD-L1 expression is typically low or absent in SCLC [17].

We conducted this real-world, multicenter, retrospective, controlled study to compare the prognosis, efficacy, and safety of PD-1 and PD-L1 inhibitors in patients with ES-SCLC when combined with chemotherapy.

## Methods

### Study Design and participants

This is a real-world, multicenter, retrospective, controlled study that included patients diagnosed with ES-SCLC who were treated in the First, Fifth, and Seventh centers of the PLA General Hospital between January 2017 and December 2021. The key eligibility criteria were as follows: (1) age ≥ 18 years; (2) histologically or cytologically confirmed ES-SCLC without prior systemic treatment; and (3) received PD-1/PD-L inhibitor plus EP chemotherapy as first-line treatment; (4) Response Evaluation Criteria in Solid Tumors (RECIST, version 1.1)-measurable diseases;. Patients who were diagnosed with limited-stage small cell lung cancer (LS-SCLC) and received EP chemotherapy alone were excluded.

Demographic and clinicopathological data were collected from the Electronic Medical Record System, including age, sex, smoking history, performance status, baseline organ metastasis, ICIs, and immunotherapy-related adverse effects (irAEs). Patient survival data were obtained through telephone follow-ups and outpatient record systems.

Due to the anticipated high rate of suboptimal sample types (such as fine-needle aspirates and bronchoscopy findings), low expression of PD-L1 on tumor cells, and unclear relationship between PD-L1 expression and immunotherapy efficacy in extensive-stage small-cell lung cancer, PD-L1 testing was not performed during screening. All patients provided written informed consent. The study protocol was approved by the Ethics Committee of PLA General Hospital (S2018-092-01).

### Treatment

Each patient received up to six cycles of etoposide (80–100 mg/m2 body surface area, intravenously on Days 1–3 of each cycle), carboplatin (area under the curve of 5–6 mg/mL/min, intravenously on Day 1 of each cycle), or cisplatin (75–80 mg/m², administered on day one of each cycle). The patients in our study received ICI drugs, including PD-1 inhibitors (pembrolizumab, nivolumab and sintilimab) and PD-L1 inhibitors (atezolizumab, durvalumab). Patients received pembrolizumab 200 mg/nivolumab 360 mg/sintilimab 200 mg or atezolizumab 1200 mg/durvalumab 1500 mg intravenously every 3 weeks after EP chemotherapy from cycle 1. After the completion of 6 cycles of EP chemotherapy, maintenance treatment was continued with PD-1 or PD-L1 inhibitors. The maximum treatment exposure to ICIs was not restricted unless disease progression, death, or intolerable toxicity occurred. Chest irradiation or brain irradiation was allowed during ICI maintenance.

### Endpoints and assessment

The primary endpoints were investigator-assessed PFS and OS. PFS was defined as the period from initiation of immunotherapy to disease progression or death from any cause, whichever occurred first. OS was defined as the time from immunotherapy initiation to death. The secondary endpoints were the investigator-assessed objective response rate (ORR), disease control rate (DCR), and duration of response (DOR) according to RECIST 1.1. ORR was defined as the proportion of patients with partial response (PR) or complete response (CR). DCR was defined as the proportion of patients showing partial response (PR), complete response (CR), or stable disease (SD).

Tumor assessment was performed by researchers from various clinical centers based on RECIST 1.1 criteria every 2 cycles initially and then every 3–4 cycles during ICI maintenance until the occurrence of disease progression or death. Immune-related adverse events (irAEs) were evaluated according to the Chinese Society of Clinical Oncology (CSCO) guidelines for the management of toxicity associated with immune checkpoint inhibitors. The researchers determined whether the adverse events were related to treatment.

All patients were followed from the initiation of first-line systemic chemo-immunotherapy until death, final recorded follow-up, or the end of the study (August 31, 2022), whichever occurred first.

### Statistical analysis

Data were analyzed using SPSS 26.0 and STATA statistical software version 17.0. The clinical demographics and safety data were summarized using descriptive statistical analyses. The differences in baseline characteristics between the PD-1 inhibitor and PD-L1 inhibitor groups were evaluated using the Wilcoxon or t test for continuous data and the Chi-square test or Fisher’s exact test for categorical data.

The primary endpoints and safety of our study were evaluated in the intention-to-treat population. PFS, OS, and DOR were described using the Kaplan‒Meier method to estimate the proportion of surviving patients. The stratified log-rank test was used to compare the PFS and OS in different subgroups. The response rates in the different subgroups were compared using the Chi-square test or Fisher’s exact test. The hazard ratios (HRs) and 95% CIs for PFS and OS were evaluated using a stratified Cox proportional hazards model. Univariate and multivariate Cox regression analyses were applied to identify independent predictors of PFS and OS.

## Results

### Patient characteristics and treatment

**Fig. 1:**
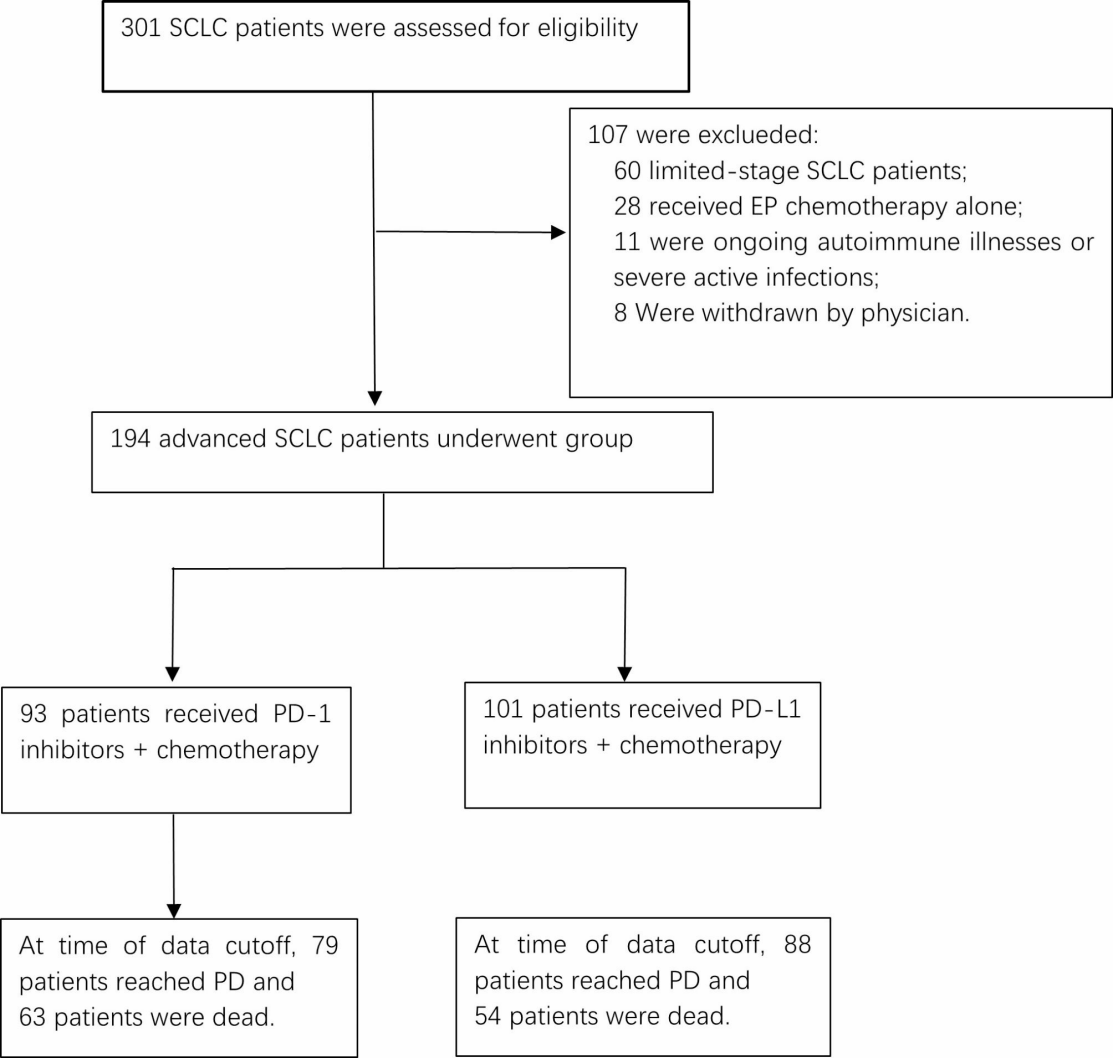
Diagram of the study. 194 advanced SCLC patients underwent group were included in the intention-to-treat (ITT) analysis. The date of data cutoff was September 28, 2022

Between January 2017 and December 2021, 301 SCLC patients from three clinical centers in a PLA general hospital were screened for eligibility, and 194 patients were finally included in the study (Fig. 1). In the intention-to-treat population, 93 patients received PD-1 inhibitor plus EP chemotherapy, and 101 patients received PD-L1 inhibitor plus EP chemotherapy (Fig. 1). The baseline clinical demographics were well balanced between the PD-1/PD-L1 inhibitor combination groups (Table 1). The median ages of the PD-1 and PD-L1 groups were 58 years (range 32–80) and 61 years (36–89), respectively. In both groups, the majority of patients were male (86% vs. 85.1%) with a history of never smoking (25.8% vs. 25.8%), current smoking (40.9% vs. 36.6%), and former smoking (33.3% vs. 37.6%). The ECOG-PS scores in the PD-1 and PD-L1 groups were 0–1 (77.4% vs. 81.2%) and 2 (22.6% vs. 18.8%), respectively. Liver metastasis, brain metastasis, and bone metastasis were observed in 26.9%, 21.5%, and 34.4%, respectively, in the PD-1 inhibitor group at baseline. These values were 22.8%, 20.8%, and 37.6%, respectively, in the PD-L1 inhibitor group at baseline. Approximately 12.9% and 8.9% of patients in the PD-1 and PD-L1 groups had four or more metastatic sites, respectively. In addition, 38.7% of patients in the PD-1 group and 45.5% of patients in the PD-L1 group received chest radiotherapy. Meanwhile, the proportions of patients who received brain radiotherapy were 18.3% and 26.7% in the PD-1/PD-L1 combination group. The baseline characteristics of all the enrolled patients are shown in Supplementary Table 1.

**Table 1:** Patients’ characteristics at baseline (intention-to-treat population)

Characteristics	PD-1 + EP group	PD-L1 + EP group
	(N = 93) (no.%)	(N = 101) (no.%)
**Median age (range)-yr**	58(32–80)	61(36–89)
**Age**		
< 65	67(72.0)	62(61.4)
≥ 65	26(28.0)	39(38.6)
**Gender**		
Female	13(14.0)	15(14.9)
Male	80(86.0)	86(85.1)
**ECOG performance status**		
0–1	72(77.4)	82(81.2)
≥ 2	21(22.6)	19(18.8)
**Smoking status** ^**a**^		
Never smoked	24(25.8)	26(25.8)
Current smoker	38(40.9)	37(36.6)
Former smoker	31(33.3)	38(37.6)
**Liver metastasis**		
No	68(73.1)	78(77.2)
Yes	25(26.9)	23(22.8)
**Brain metastasis**		
No	73(78.5)	80(79.2)
Yes	20(21.5)	21(20.8)
**Bone metastasis**		
No	61(65.6)	63(62.4)
Yes	32(34.4)	38(37.6)
**Number of metastasis sites**		
1–3	81(87.1)	92(91.1)
≥4	12(12.9)	9(8.9)
**Chest raditherapy**		
No	57(61.3)	55(54.5)
Yes	36(38.7)	46(45.5)
**Brain radiotherapy**		
N0	76(81.7)	74(73.3)
Yes	17(18.3)	27(26.7)
**Serum LDH (U/L)**		
Median (range)	216(112–1750)	232(114–1506)

*Abbreviations*: *ECOG* Eastern Cooperative Oncology Group; *LDH* lactic dehydrogenase; *PD-1* programmed death protein-1; *PD-L1* programmed death-ligand 1; *EP* platinum-etoposide

^a^A current smoker is someone who smokes or hasn’t quit for less than a year; a former smoker is someone who hasn’t smoked in more than a year; a never smoker is someone who has never smoked more than 100 cigarettes in their life

### Survival outcomes

**Fig. 2:**
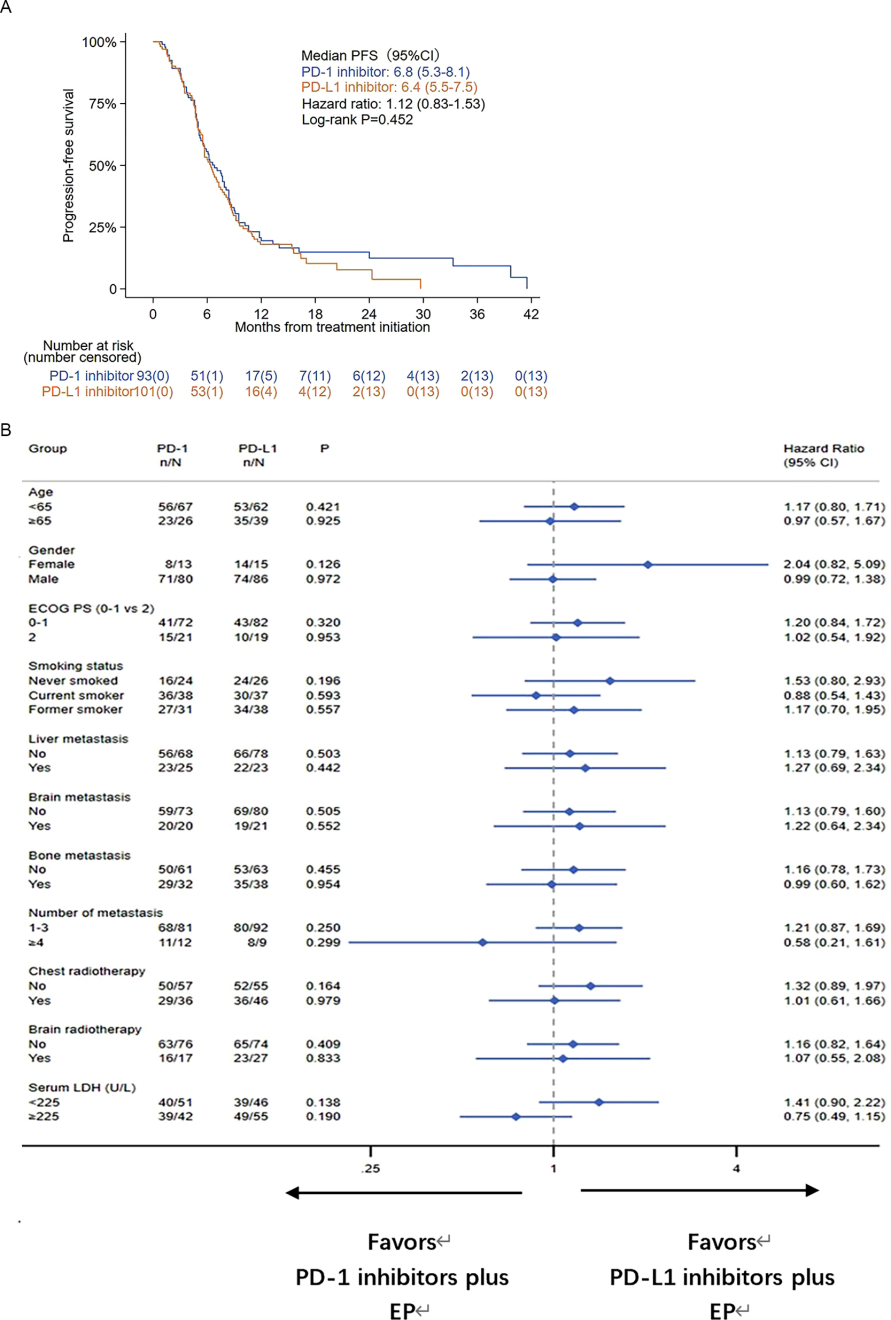
(**A**) Kaplan-Meier curve of progression-free survival (PFS) in the intention-to-treat population. (**B**) Forest plot of subgroup analysis of PFS in the intention-to-treat population

At the time of data cutoff, the median follow-up for all patients was 18.6 months. There were 117 (60%) deaths across the intention-to-treat population: 63 patients (67.7%) in the PD-1 inhibitor group and 54 (53.5%) patients in the PD-L1 inhibitor group had died. A total of 79 patients (84.9%) in the PD-1 inhibitor group and 88 patients (87.1%) in the PD-L1 inhibitor group experienced disease progression or died. Progression-free survival in the PD-1 group (median PFS, 6.8 months; 95% CI, 5.3–8.1) was similar to that in the PD-L1 group (median PFS, 6.4 months; 95% CI, 5.5–7.5) (Fig. 2A). The stratified hazard ratio for PFS was 1.12 (95% CI, 0.83–1.53; P = 0.452). The 6-month progression-free survival estimate rates were 49% versus 45%, and the 12-month progression-free survival estimate rates were 12% versus 18%.

The median OS was similar in the PD-1 inhibitor and PD-L1 inhibitor groups (15.8 m vs. 17.7 m, P = 0.566) (Fig. 2B). The stratified hazard ratio for OS was 0.90 (95% CI, 0.62–1.30, P = 0.566). The estimated 12-month overall survival rates were 57% in the PD-1 group and 60% in the PD-L1 group. The estimated 18-month overall survival rates were 43% and 49% in the PD-1 and PD-L1 groups, respectively.

**Fig. 3:**
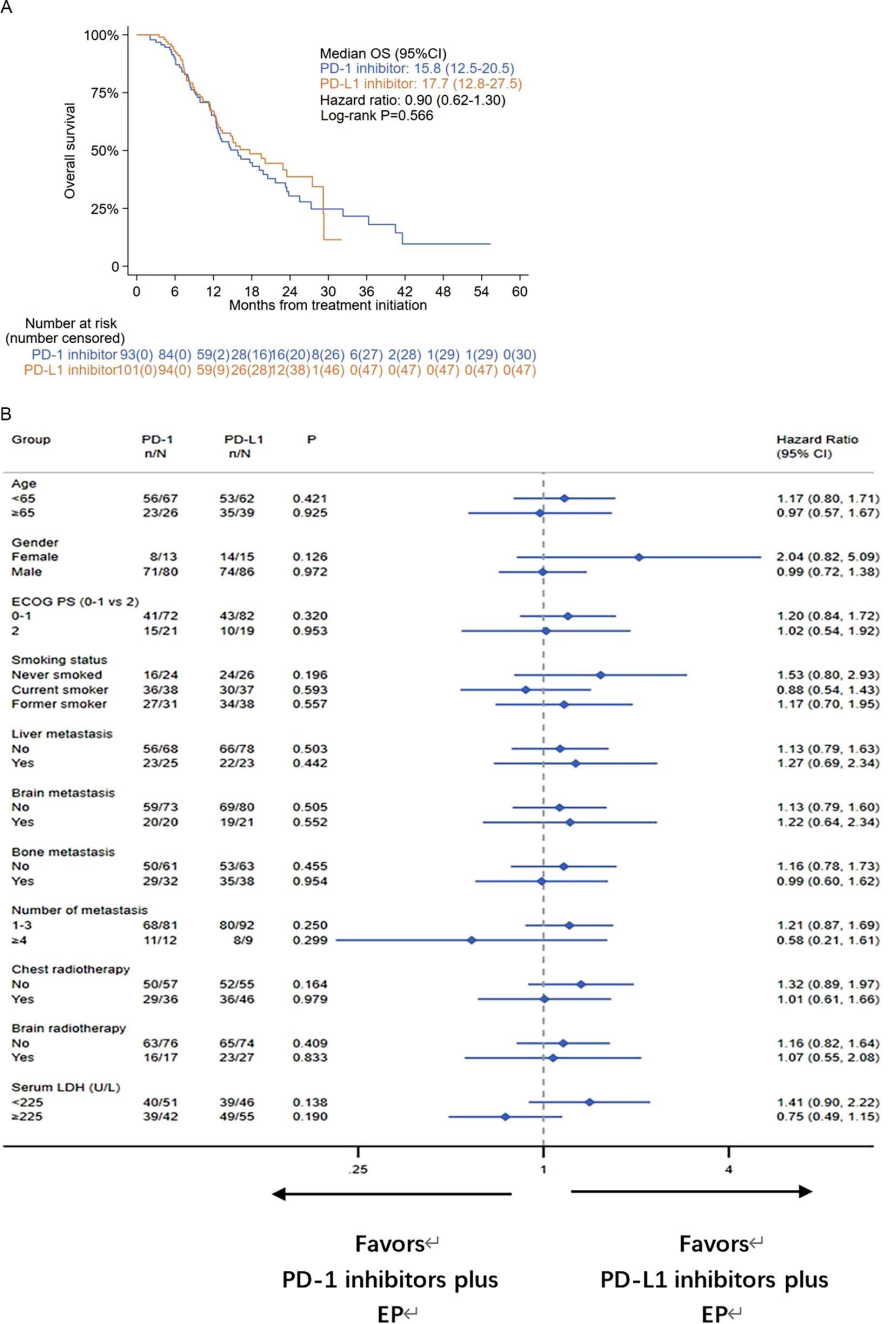
(**A**) Kaplan-Meier curve of overall survival (OS) in the intention-to-treat population. (**B**) Forest plot of subgroup analysis of OS in the intention-to-treat population

We conducted an exploratory subgroup analysis stratified by age, sex, ECOG PS, smoking status, liver metastasis, brain metastasis, bone metastasis, number of metastases, chest radiotherapy, brain radiotherapy, and serum LDH. In the subgroup analysis, no significant differences were observed between the PD-1 inhibitor and PD-L1 inhibitor groups in terms of PFS and OS (Fig. 3A, B). Univariate and multivariate analyses for PFS and OS according to the baseline characteristics of all patients are shown in Supplementary Tables 2 and 3.

### Activity

The two groups exhibited comparable investigator-assessed confirmed objective response rates and median duration of response (Table 2); 71 (76.3%) patients had an objective response rate in the PD-1 inhibitor plus EP group compared to 77 (76.2%) patients in the PD-L1 inhibitor plus EP group. Unfortunately, none of the patients achieved complete remission in our study. Among responders, the median duration of response was 6.2 months (95% CI, 1.4–38.6) in the PD-1 group and 6.1 months (95% CI, 1.4–28.3) in the PD-L1 group. At the time of data cutoff, the ongoing response was the same for both groups (16.9% vs. 16.9%).

**Table 2:** Response rates according to checkpoint-inhibitor strategy in advanced SCLC patients

	PD-1 group	PD-L1 group
	(N = 93)	(N = 101)
**Best response**		
Partial response-n (%)	71 (76.3)	77 (76.2)
Stable disease-n (%)	14 (15.1)	14 (13.9)
Progressive disease-n (%)	8 (8.6)	10 (9.9)
**Disease control rate- n (%)**	79 (91.4)	87 (90.1)
**Ongoing response at data cutoff — no./total no. (%)**	12/71 (16.9)	13/77 (16.9)
**Median duration of response (range)- mo**	6.2 (1.4–38.6)	6.1 (1.4–28.3)

### Safety

Treatment-related adverse events (TRAEs) are summarized in Table 2. Adverse events related to any cause or grade occurred in 77 (82.7%) patients who received PD-1 inhibitor plus EP chemotherapy and 86 (85.1%) patients who received PD-L1 inhibitor plus EP chemotherapy, including 31 (33.3%) and 34 (33.7%) patients who experienced grade 3 or 4 adverse events (AEs), respectively. AE-related discontinuation occurred in 4 (4.3%) patients in the PD-1 inhibitor group and 2 (2.0%) patients in the PD-L1 inhibitor group. Deaths due to AEs of any cause occurred in 2 (2.2%) patients in the PD-1 inhibitor group and 1 (1.0%) patient in the PD-L1 inhibitor group. In both groups, the most common TRAEs of any grade were anemia, neutropenia, and febrile neutropenia in the PD-1 group, while anemia, neutropenia, and fatigue were the most common grade 3–4 AEs in the PD-L1 group (Table 3).

**Table 3:** Adverse events related to the treatment

	PD-1 inhibitor plus		PD-L1 inhibitor plus	
	EP group(n = 93)		EP group(n = 101)	
	Any grade	Grade3-4	Any grade	Grade3-4
**Treatment-related adverse events**	77(82.7%)	31(33.3%)	86(85.1%)	34(33.7%)
**Any event leading to discontinuation**	4(4.3%)		2(2.0%)	
**Any event leading to death**	2(2.2%)		1(1.0%)	
Fatigue	30(32.3%)	1(1.1%)	32(31.6%)	
Neutropenia	56(60.2%)	19(20.4%)	62(61.3%)	23(22.8%)
Febrile neutropenia	8(8.6%)	8(8.6%)	11(11%)	11(10.9%)
Thrombocytopenia	20(21.5%)	7(7.5%)	20(19.8%)	7(6.9%)
Vomiting	21(22.5%)	1(1.1%)	19(18.8%)	
Nausea	24(25.8%)	1(1.1%)	20(19.8%)	
Anemia	49(52.6%)	16(17.2%)	45(44.6%)	16(15.8%)
Alopecia	4(4.3%)		13(12.9%)	2(2.0%)
Constipation	11(11.8%)		18(17.8%)	
Colitis	1(1.1%)		1(1.0%)	
Creatinine elevation	1(1.1%)		1(1.0%)	1(1.0%)
**Immune-related adverse events**	23(24.7%)	5(5.4%)	33(32.7%)	5(5.0%)
Skin toxicity	9(10%)		10(10.0%)	
lung toxicity	11(12%)	2(2.2%)	14(14%)	4(4.0%)
Gastrointestinal toxicity	2(2.2%)		5(5.0%)	
Liver/Pancreas toxicity	4(4.3%)	2(2.2%)	4(4.0%)	
Endocrine toxicity	2(2.2%)	1(1.1%)	3(3.0%)	1(1.0%)
Neurological toxicity	2(2.2%)		2(2.0%)	
Other	1(1.1%)		2(2.0%)	

Immunotherapy-related adverse events (irAEs) were reported in 23 (24.7%) patients in the PD-1 inhibitor group and 33 (32.7%) patients in the PD-L1 inhibitor group, including 5 (5.4%) and 5 (5.0%) patients with grade 3–4 irAEs, such as pneumonitis and liver/pancreatic toxicity, respectively. The most common irAEs of any grade were skin rash and lung toxicity.

## Discussion

Currently, there are numerous real-world studies reporting on the comparison between first-line immunotherapy combined with chemotherapy and stand-alone chemotherapy for small-cell lung cancer [20–22]. These real-world studies demonstrated that in comparison with chemotherapy alone, the addition of PD-1/PD-L1 inhibitors to chemotherapy as first-line treatment for ES-SCLC significantly improves both the PFS and OS, without increasing adverse events.

The findings of our research suggest that there is no difference in outcomes between anti-PD-1 and anti-PD-L1 agents in combination with chemotherapy in ES-SCLC patients. A manageable safety profile was observed in both the PD-1 and PD-L1 inhibitor groups, with a low rate of treatment termination due to adverse events. To the best of our knowledge, this is the first multicenter, real-world study analyzing the differences between PD-1 inhibitor plus EP chemotherapy and PD-L1 inhibitor plus EP chemotherapy on survival, efficacy, and safety in ES-SCLC patients from China.

Several global, randomized, phase 3 clinical trials have demonstrated the advantage of immune checkpoint inhibitors (ICIs) in combination with chemotherapy as the first-line treatment for ES-SCLC. The IMpower133 study [12, 23] showed that atezolizumab plus EP chemotherapy significantly prolonged progression-free survival (PFS) (HR 0.77, 95% CI 0.62–0.96, P = 0.02) and overall survival (OS) (HR 0.70, 95% CI 0.54–0.91, P = 0.007) versus chemotherapy alone, with a median PFS of 5.2 vs. 4.3 months and median OS of 12.3 vs. 10.3, respectively. The CASPIAN clinical trial [13, 24] demonstrated that another PD-L1 inhibitor, durvalumab combined with chemotherapy, also provided a distinct improvement in OS (13.0 vs. 10.3 months, HR 0.73, 95% CI 0.59–0.91, P = 0.0047). Recently, the CAPSTONE-1 study found that adebrelimab, a novel anti-PD-L1 antibody, exhibited a median OS improvement of 2.5 months in the immunotherapy plus chemotherapy group (median, 15.3 vs. 12.8 months, HR: 0.72, 95% CI: 0.58–0.90) in a Chinese population [25].

The promising antitumor activity of PD-1 inhibitors combined with chemotherapy has been shown for non-small cell lung cancer (NSCLC) in several randomized controlled trials (RCTs), but data on PD-1 inhibitors plus chemotherapy are relatively lacking in SCLC patients. The KEYNOTE-604 study [14] verified that adding pembrolizumab to EP chemotherapy significantly improved PFS (HR: 0.75, 95% CI: 0.61–0.91; P = 0.0023) compared with placebo plus EP as first-line therapy for ES-SCLC but not OS (HR, 0.80; 95% CI, 0.64–0.98; P = 0.0164). Although the significance level was not satisfactory, OS was distinctively prolonged with pembrolizumab plus EP in this study. The ASTRUM-005 randomized clinical trial demonstrated that serplulimab plus chemotherapy significantly increased overall survival compared to chemotherapy alone in patients with previously untreated extensive-stage SCLC (15.4 vs. 10.9 months, HR = 0.63, P < 0.001) as well as progression-free survival (5.7 vs. 4.3 months, HR = 0.48) [11]. Serplulimab was the first PD-1 inhibitor that was approved by NMPA in combination with chemotherapy for first-line treatment of ES-SCLC, reshaping the first-line treatment landscape of ES-SCLC in China.

Several meta-analyses have compared the clinical benefits and safety of PD-1 inhibitor + chemotherapy and PD-L1 inhibitor + chemotherapy regimens [17, 26, 27]. Hui Yu et al. found no statistically significant changes in PFS, OS, or ORR for ES-SCLC between PD-1 + chemotherapy and PD-L1 + chemotherapy. However, PD-L1 with chemotherapy demonstrated a statistically better safety profile in lowering the chance of treatment discontinuation owing to adverse events [17]. Shuo Kang et al. [27] conducted a network meta-analysis (NMA) to compare the efficacy and evaluate the cost-effectiveness of PD-1 plus chemotherapy and PD-L1 plus chemotherapy as first-line treatments for ES-SCLC from the perspective of the Chinese health care system. The research has shown that nivolumab plus chemotherapy could bring the greatest clinical benefit, and atezolizumab plus chemotherapy is indicated as a cost-effective option in comparison to other first-line regimens for ES-SCLC [27]. There are no large clinical trials comparing the efficacy and safety of different immunotherapies directly, so the level of evidence for this type of comparison is restricted.

Compared with the IMpower133, CASPIAN, and KEYNOTE604 study populations, the patients in our study were slightly younger (median age 60 vs. 62–65 years), and there was a lower proportion of baseline liver metastasis (21% vs. 40%) as well as ≥ 3 metastatic sites (35% vs. 61%). The proportion of never smokers was higher in our study. In randomized, phase 3 clinical trials, patients in the immunotherapy group received up to four cycles of EP chemotherapy with atezolizumab, durvalumab, or pembrolizumab, whereas patients in our study received up to six cycles of ICIs plus chemotherapy. In addition, there was a higher ORR in our study compared to IMpower133 (76% vs. 60%), which implied that immunotherapy plus chemotherapy may have a better benefit in Asian SCLC populations. Notably, compared to the IMpower133, CASPIAN, and KEYNOTE-604 studies, subsequent systemic antitumor therapy was administered more frequently in the treatment of Chinese patients.

In particular, antiangiogenic therapy, such as anlotinib [28] and apatinib [29], and vascular endothelial growth factor receptor (VEGFR) inhibitors are widely used as third-line or further treatment of SCLC. Furthermore, the percentage of patients who received chest radiotherapy or brain radiotherapy in our study was obviously higher than that in other phase 3 clinical trials, which resulted in longer PFS and OS. Based on the above reasons, the median overall survival in our study (15.8 vs. 17.7 months) was longer than that in other studies (9·7–10·3 months) [12–14] but similar to the CAPSTONG-1 results (median OS: 15.3 months in the immunotherapy group) [25]. The median overall survival was 2 months longer in the PD-L1 group than in the PD-1 group, but the differences between the two groups were not statistically significant in our study. Furthermore, the safety profiles in the PD-1 and PD-L1 groups were generally similar, but any grade of treatment-related adverse event occurred with slightly higher frequency in the PD-L1 group, and most were grade 1–2 AEs. Events leading to discontinuation or death in the two groups were too few to compare.

The accuracy of PD-L1 expression in predicting the effectiveness of PD-L1/PD-1 + Chemo in SCLC is low, although it has been considered a possible prognostic biomarker of response to ICIs in various tumor types [14, 23]. One explanation for this is that SCLC biopsy samples are frequently small and heavily composed of necrotic tissue. Another reason is that unlike NSCLC, SCLC largely expresses PD-L1 on tumor-infiltrating immune cells (ICs) rather than tumor cells (TCs). These results indicate that PD-L1 expression may not be a useful biomarker for predicting the response to PD-L1 or PD-1 plus chemotherapy in SCLC patients [17, 30]. Another hallmark of the immunological microenvironment for SCLC is the tumor mutational burden (TMB). However, in IMpower133, the prognostic value of TMB was examined only among the four studies that were included, and results could not be pooled [17].

Therefore, markers other than TMB and PD-L1 are needed in SCLC, such as the molecular subtypes proposed by Gay et al. [31] and Rudin et al. [32]. In patients with SCLC, elevated LDH is regarded as a poor prognostic indicator, foretelling a poor response to ICIs [33, 34]. In our study, increased LDH levels were also associated with worse OS. Further research is warranted to unveil the value of biomarkers for immunotherapy in SCLC.

This study had several limitations. First, this was a retrospective analysis, and baseline characteristics as well as treatment-related adverse events were collected from electronic medical records, which were constrained by the accuracy of treating physicians’ documentation. Second, due to the COVID-19 epidemic, patients from different cities could not come to Beijing for treatment and chose to receive corresponding treatment in local hospitals, resulting in recall bias and partial loss of follow-up data. Third, baseline data on PD-L1 expression and TMB were not collected; hence, whether PD-L1 expression and TMB could be predictive biomarkers for efficacy or prognosis in ES-SCLC is unknown. Fourth, the patients included in our study selected PD-1 monoclonal antibodies from domestic and abroad and different pharmaceutical manufacturers, possibly leading to bias. Fifth, the affordability of specific drugs could have affected the results. Patients who can afford PD-L1 drugs tend to have higher social status and better financial status—enough to support their subsequent antitumor therapy, and ultimately, these patients tend to have better survival. Finally, all patients included in our research were from China, and the differences between the PD-1 and PD-L1 groups in patients of other ethnicities remain to be confirmed. Despite these limitations, we believe that the present study reflects the real-world clinical conditions of ES-SCLC patients in China. Subsequent studies with head-to-head randomized controlled trials are needed.

## Conclusions

Our study revealed no significant differences in efficacy and prognosis between PD-1 inhibitor + EP chemotherapy and PD-L1 inhibitor + EP chemotherapy. The two groups seemed to have comparable safety profiles, but the event numbers of discontinuation or death were too small to draw a firm conclusion. Clinically, our study may aid clinicians in decision-making and provide other options for patients with ES-SCLC who cannot afford PD-L1 inhibitors.

### Electronic supplementary material

Below is the link to the electronic supplementary material.

Supplementary Material 1

## Data Availability

Data are accessible with reasonable request. All related data are included in the article and in additional online files or are available from the corresponding author upon reasonable request.
