# Supplementary material for: Comparison of efficacy and safety between PD-1 inhibitors and PD-L1 inhibitors plus platinum-etoposide as first-line treatment for extensive-stage small-cell lung cancer: a multicenter, real-world analysis
Source: BMC Cancer. 2023 Dec 6;23:1196. doi: 10.1186/s12885-023-11709-1 (PMC10701967; doi:10.1186/s12885-023-11709-1)
Supplement: Supplementary file 1 — Supplementary Material 1 [file 12885_2023_11709_MOESM1_ESM.docx]

Supplementary Material

**Comparison of efficacy and safety between PD-1 inhibitors and PD-L1 inhibitors plus platinum-etoposide as first-line treatment for extensive-stage small-cell lung cancer: a multicenter, real-world analysis**

**Yanrong Wang^1,2†^, Lingling Li^5†^, Jia Hu^3,4†^, Yan Zhao^3^, Huan Yan^3^, Ming Gao^3^, Xuejiao Yang^3^, Xia Zhang^3*^, Junxun Ma^3*^, Guanghai Dai^3*^**

1. **Department of Medical Oncology, the First Medical Center, Chinese PLA General Hospital, Beijing, China**
2. **Chinese PLA Medical School, Beijing, China**
3. **Department of Medical Oncology, the Fifth Medical Center, Chinese PLA General Hospital, Beijing, China**
4. **Department of Medical Oncology, the Seventh Medical Center, Chinese PLA General Hospital, Beijing, China**
5. **School of Medicine, Nankai University, Tianjin, China**

**^*^Corresponding author:**  Xia Zhang, Email: [zx6055215@163.com](mailto:zx6055215@163.com); Junxun Ma, Email: [majunxun74@126.com](mailto:majunxun74@126.com); Guanghai Dai, Email: [dgh19661007@126.com](mailto:dgh19661007@126.com)

**^†^**These authors have contributed equally to this work and share first authorship.

# Supplementary Tables

**Supplementary Table 1 Baseline Characteristics of All Enrolled Patients**

| **Characteristics** | **No. of patients(N=194)** | **Percentage (%)** |
| --- | --- | --- |
| **Median age (range)-yr** | 60 (32-89) |  |
| **Age** |  |  |
| <65 | 129 | 66.5 |
| ≥65 | 65 | 33.5 |
| **Gender** |  |  |
| Female | 28 | 14.4 |
| Male | 166 | 85.6 |
| **ECOG performance status** |  |  |
| 0-1 | 154 | 79.4 |
| ≥2 | 40 | 20.6 |
| **Smoking status** |  |  |
| Never smoked | 50 | 25.8 |
| Current smoker | 75 | 38.7 |
| Former smoker | 69 | 35.6 |
| **Liver metastasis** |  |  |
| No | 146 | 75.3 |
| Yes | 48 | 24.7 |
| **Brain metastasis** |  |  |
| No | 153 | 78.9 |
| Yes | 41 | 21.1 |
| **Bone metastasis** |  |  |
| No | 124 | 63.9 |
| Yes | 70 | 36.1 |
| **Number of metastasis sites** |  |  |
| 1-3 | 173 | 89.2 |
| ≥4 | 21 | 10.8 |
| **ICIs drugs** |  |  |
| PD-1 inhibitors | 93 | 47.7 |
| PD-L1 inhibitors | 101 | 52.3 |
| **Chest raditherapy** |  |  |
| No | 112 | 57.7 |
| Yes | 82 | 42.3 |
| **Brain radiotherapy** |  |  |
| N0 | 150 | 77.3 |
| Yes | 44 | 22.7 |
| **Serum LDH (U/L)** |  |  |
| Median (range) | 225 (112-1750) |  |

**Supplementary Table 2 Univariate and Multivariate analyses for PFS according to baseline characteristics in all patients**

| **Subgroup** | **Univariate analysis** | | |  |  | **Multivariate analysis** | | | |
| --- | --- | --- | --- | --- | --- | --- | --- | --- | --- |
|  | **HR** | **95%CI** | **P value** | |  | **HR** | **95%CI** | **P value** | |
| **Age** |  |  | 0.360 | |  |  |  | 0.403 |  |
| <65 | 1.0 |  |  |  |  | 1.0 |  |  |  |
| ≥65 | 1.16 | 0.84-1.60 |  |  |  | 1.16 | 0.82-1.62 |  |  |
| **Gender** |  |  | 0.194 | |  |  |  | 0.166 |  |
| Female | 1.0 |  |  |  |  | 1.0 |  |  |  |
| Male | 1.35 | 0.86-2.12 |  |  |  | 0.61 | 0.30-1.23 |  |  |
| **ECOG PS** |  |  | 0.000 | |  |  |  | 0.002 |  |
| 0-1 | 1.0 |  |  |  |  | 1.0 |  |  |  |
| ≥2 | 2.70 | 1.87-3.91 |  |  |  | 2.05 | 1.30-3.26 |  |  |
| **Smoking status** |  |  | 0.329 | |  |  |  | 0.036 |  |
| Never smoked | 1.0 |  |  |  |  | 1.0 |  |  |  |
| Current smoker | 1.33 | 0.90-1.98 | 0.154 |  |  | 2.08 | 1.19-3.64 | 0.01 |  |
| Former smoker | 1.28 | 0.86-1.92 | 0.224 |  |  | 1.76 | 1.00-3.10 | 0.05 |  |
| **Liver metastasis** |  |  | 0.000 | |  |  |  | 0.027 |  |
| No | 1.0 |  |  |  |  | 1.0 |  |  |  |
| Yes | 2.36 | 1.66-3.35 |  |  |  | 1.64 | 1.06-2.54 |  |  |
| **Brain metastasis** |  |  | 0.285 |  |  |  |  | 0.393 |  |
| No | 1.0 |  |  | |  | 1.0 |  |  |  |
| Yes | 1.22 | 0.85-1.75 |  |  |  | 1.20 | 0.79-1.83 |  |  |
| **Bone metastasis** |  |  | 0.000 | |  |  |  | 0.295 |  |
| No | 1.0 |  |  |  |  | 1.0 |  |  |  |
| Yes | 1.81 | 1.32-2.49 |  |  |  | 1.22 | 0.84-1.78 |  |  |
| **Number of metastasis** | |  | 0.001 | |  |  |  | 0.721 |  |
| 1-3 | 1.0 |  |  |  |  | 1.0 |  |  |  |
| ≥4 | 2.32 | 1.42-3.78 |  |  |  | 0.90 | 0.50-1.62 |  |  |
| **ICIs drugs** |  |  | 0.455 | |  |  |  | 0.255 |  |
| PD-1 inhibitors | 1.0 |  |  |  |  | 1.0 |  |  |  |
| PD-L1 inhibitors | 1.13 | 0.83-1.53 |  |  |  | 1.21 | 0.87-1.69 |  |  |
| **Chest radiotherapy** |  |  | 0.000 | |  |  |  | 0.025 |  |
| No | 1.0 |  |  |  |  | 1.0 |  |  |  |
| Yes | 0.51 | 0.37-0.70 |  |  |  | 0.65 | 0.45-0.95 |  |  |
| **Brain radiotherapy** |  |  | 0.483 |  |  |  |  | 0.975 |  |
| No | 1.0 |  |  |  |  | 1.0 |  |  |  |
| Yes | 0.88 | 0.61-1.26 |  |  |  | 0.99 | 0.66-1.51 |  |  |
| **Serum LDH (U/L)** |  |  | 0.000 | |  |  |  | 0.36 |  |
| <225 | 1.0 |  |  |  |  | 1.0 |  |  |  |
| ≥225 | 1.80 | 1.34-2.46 |  |  |  | 1.20 | 0.81-1.77 |  |  |

**Supplementary Table 3 Univariate and Multivariate analyses for OS according to baseline characteristics in all patients**

| **Subgroup** | |  | | **Univariate analysis** | | |  | **Multivariate analysis** | | |  | |
| --- | --- | --- | --- | --- | --- | --- | --- | --- | --- | --- | --- | --- |
|  | | **HR** | **95%CI** | |  | **P value** |  | **HR** | **95%CI** | **P value** | |  |
| **Age** |  | |  | |  | 0.133 |  |  |  | 0.218 |  | |
| <65 | 1.0 | |  | |  |  |  | 1.0 |  |  |  | |
| ≥65 | 1.34 | | 0.92-1.95 | |  |  |  | 1.29 | 0.86-1.94 |  |  | |
| **Gender** |  | |  | |  | 0.199 |  |  |  | 0.190 |  | |
| Female | 1.0 | |  | |  |  |  | 1.0 |  |  |  | |
| Male | 1.43 | | 0.83-2.46 | |  |  |  | 0.55 | 0.23-1.42 |  |  | |
| **ECOG PS** |  | |  | |  | 0.000 |  |  |  | 0.000 |  | |
| 0-1 | 1.0 | |  | |  |  |  | 1.0 |  |  |  | |
| ≥2 | 5.16 | | 3.42-7.79 | |  |  |  | 3.47 | 2.08-5.78 |  |  | |
| **Smoking status** |  | |  | |  | 0.281 |  |  |  | 0.004 |  | |
| Never smoked | 1.0 | |  | |  |  |  | 1.0 |  |  |  | |
| Current smoker | 1.42 | | 0.89-2.26 | |  | 0.144 |  | 2.90 | 1.43-5.89 | 0.003 |  | |
| Former smoker | 1.11 | | 0.68-1.82 | |  | 0.683 |  | 1.77 | 0.84-3.70 | 0.132 |  | |
| **Liver metastasis** |  | |  | |  | 0.000 |  |  |  | 0.032 |  | |
| No | 1.0 | |  | |  |  |  | 1.0 |  |  |  | |
| Yes | 3.51 | | 2.38-5.17 | |  |  |  | 1.69 | 1.05-2.73 |  |  | |
| **Brain metastasis** |  | |  | |  | 0.897 |  |  |  | 0.614 |  | |
| No |  | |  | |  |  |  | 1.0 |  |  |  | |
| Yes | 0.97 | | 0.63-1.49 | |  |  |  | 1.15 | 0.67-1.97 |  |  | |
| **Bone metastasis** |  | |  | |  | 0.000 |  |  |  | 0.019 |  | |
| No | 1.0 | |  | |  |  |  | 1.0 |  |  |  | |
| Yes | 2.92 | | 2.01-4.25 | |  |  |  | 1.68 | 1.09-2.58 |  |  | |
| **Number of metastasis** |  | |  | |  | 0.000 |  |  |  | 0.046 |  | |
| 1-3 | 1.0 | |  | |  |  |  | 1.0 |  |  |  | |
| ≥4 | 3.92 | | 2.37-6.47 | |  |  |  | 1.84 | 1.01-3.34 |  |  | |
| **ICIs drugs** |  | |  | |  | 0.568 |  |  |  | 0.354 |  | |
| PD-1 inhibitors | 1.0 | |  | |  |  |  | 1.0 |  |  |  | |
| PD-L1 inhibitors | 0.90 | | 0.62-1.30 | |  |  |  | 0.83 | 0.56-1.23 |  |  | |
| **Chest radiotherapy** |  | |  | |  | 0.000 |  |  |  | 0.575 |  | |
| No | 1.0 | |  | |  |  |  | 1.0 |  |  |  | |
| Yes | 0.45 | | 0.31-0.67 | |  |  |  | 0.87 | 0.55-1.40 |  |  | |
| **Brain radiotherapy** |  | |  | |  | 0.001 |  |  |  | 0.014 |  | |
| No | 1.0 | |  | |  |  |  | 1.0 |  |  |  | |
| Yes | 0.44 | | 0.27-0.73 | |  |  |  | 0.49 | 0.28-0.87 |  |  | |
| **Serum LDH (U/L)** |  | |  | |  | 0.000 |  |  |  | 0.013 |  | |
| <225 | 1.0 | |  | |  |  |  | 1.0 |  |  |  | |
| ≥225 | 2.84 | | 1.93-4.18 | |  |  |  | 1.81 | 1.13-2.88 |  |  | |
